# Growth hormone deficiency and replacement therapy in adults: Impact on survival

**DOI:** 10.1007/s11154-020-09599-w

**Published:** 2020-10-17

**Authors:** Christa C. van Bunderen, Daniel S. Olsson

**Affiliations:** 1 Department of Internal Medicine, Sub-section of Endocrinology, Vrije Universiteit Amsterdam, Amsterdam UMC, Amsterdam, 1117 The Netherlands; 2 Division of Endocrinology, Department of Internal Medicine, Radboud University Medical Center, Nijmegen, 6525 GA The Netherlands; 3 Department of Endocrinology, Sahlgrenska University Hospital, 413 45 Gothenburg, Sweden; 4 Department of Internal Medicine and Clinical Nutrition, Institute of Medicine, The Sahlgrenska Academy, University of Gothenburg, 405 30 Gothenburg, Sweden

**Keywords:** Growth hormone, Growth hormone deficiency, Growth hormone replacement therapy, Mortality, Survival

## Abstract

In a seminal paper from 1990, Rosen and Bengtsson suggested that hypopituitary patients with a presumed growth hormone (GH) deficiency (GHD) have an excess mortality. Later studies have confirmed this finding but have also shown that the cause of the increased risk of death in these patients is multifactorial, including unreplaced GHD as well as non-physiological replacement therapy of other deficiencies, the etiology of hypopituitarism, and the side effects of tumor treatment. Only a few studies have investigated mortality in hypopituitary patients with GHD receiving GH replacement therapy (GHRT): these studies are retrospective observational studies with a wide range of underlying diseases but most of them show a mortality that is not different from the general population. Even though the research field of survival in GHD patients with and without GHRT is lacking prospective randomized trials, the evidence suggests that GHD in hypopituitary patients contributes to an excess mortality and modern replacement therapy including GHRT will result in a mortality that is approaching normal. Herein, we review the literature in the field of survival in GHD patients with and without GHRT. In addition, we outline the most important issues when evaluating studies in this area.

## Introduction

Growth hormone (GH) deficiency (GHD) in adults is a well-known entity for several decades. Early reports and hypotheses on the effects of GHD in adults were already formulated at the start of the 1960s [1, 2], but it was not until the availability of recombinant human GH that the research field became firmly established. Today, we know that adult GHD is characterized by abnormal body composition, reduced muscle strength and exercise capacity, reduced bone mass, an adverse cardiovascular risk profile, and impaired quality of life [3, 4].

In a seminal article in 1990, Rosen and Bengtsson showed that patients with hypopituitarism including an assumed GHD, but substituted for all other deficiencies except GHD, had an excess mortality [5]. This finding of excess mortality in patients with unreplaced GHD has later been verified by several studies [6, 7]. Hypopituitarism and its underlying causes are complex and, today, it is believed that the reason for the excess mortality is multifactorial including, for example, the etiology of hypopituitarism, GHD, non-physiological (glucocorticoid) replacement therapies, and tumor treatment and its side effects [8–10]. This role for factors other than GHD has been confirmed by the West Midlands Prospective Hypopituitary Study, since it did not find an association between the degree of hypopituitarism and mortality but did find an association with the risk factors mentioned above [10].

The effect of daily GH replacement therapy (GHRT) was first shown in two placebo-controlled studies in 1989 [11, 12]. Since then, many studies have shown that long-term daily GHRT ameliorates or reverses many of the effects of GHD, including beneficial effects on muscle and fat mass, lipid profile, diastolic blood pressure, and bone mass as well as improving quality of life [13–16]. A few retrospective observational studies have suggested that mortality is normalized in patients with GHD receiving modern replacement therapy including GHRT [17–19].

The effect of GHD on mortality is not as clear cut as once perceived. In addition, data regarding the effect of GHRT on mortality is still scarce. Therefore, the aim of this paper is to review the effect of severe GHD and daily GHRT on survival in adult patients.

## GHD and mortality

The first study that demonstrated an excess mortality in adults with hypopituitarism and presumed (untreated) GHD was published by Rosen and Bengtsson in 1990 [5]. They studied 333 patients with hypopituitarism and compared mortality to that of the general population. A total of 104 deaths were observed in patients compared to 57.4 expected (*P* < 0.001), with women tending to have a higher mortality than men. Mortality due to vascular diseases was most common in patients (60 vs. 30.8 expected, *P* < 0.001), which was unrelated to sex, age, duration of disease, radiotherapy, diabetes, blood pressure, or calendar year of treatment. Patients treated for Cushing’s disease or acromegaly were excluded because of their inherent excess mortality. All patients were suspected as having GHD; however, only 53 of the 333 were properly tested but, of these, all were proven to have severe GHD. The authors’ hypothesis was that the excess mortality was caused by untreated GHD. Subsequently, four more studies have been published on this issue, namely adult hypopituitarism including suspected GHD and mortality (see Table 1) [6, 7, 10, 20].

**Table 1:** Overview of the articles published on mortality and hypopituitarism without growth hormone replacement therapy

Reference and country	Etiology	Study population (no.) and mean age*	Controls	Follow-up duration†	Conclusions
Rosen & Bengtsson (1990) [5] Sweden	Hypopituitarism including 10% craniopharyngioma; Acromegaly + CD excluded; GHD 16% tested, of which 100% GHD	333 (W 129, M 204) 43.1/48.6 yr	General population	± 30 yr	All-cause death rate: 104 vs. 57.4 (*P* < 0.001) W: 41 vs. 14.5, M: 63 vs. 42.9; CV death rate: 60 vs. 30.8 (*P* < 0.001) W: 20 vs. 7.4, M: 40 vs. 23.5; Malignancy death rate: 7 vs. 14.2 W: 4 vs. 4.1 (*P =* 0.056), M: 3 vs. 10.1 (*P* < 0.02)
Bates et al. (1996) [7] UK	Hypopituitarism including 13% extrapituitary tumor; Acromegaly + CD excluded; GHD 55% tested, of which 96% GHD	172 (W 70, M 102) 52 yr	General population	NR	All-cause mortality SMR: 1.73 (95% CI 1.28–2.18) W: 2.29 (95% CI 1.37–3.58), M: 1.50 (95% CI 1.02–2.13); CV mortality SMR: 1.35 (95% CI 0.84–2.07) W: 1.46 (95% CI 0.50–3.20, ), M: 1.32 (95% CI 0.74–2.17); Malignancy mortality SMR: 1.41 (95% CI 0.73–2.47) W: 3.21 (95% CI 1.46–6.11), M: 0.53 (95% CI 0.11–1.54)
Bulow et al. (1997) [6] Sweden	Hypopituitarism including 12% craniopharyngioma; Acromegaly + CD excluded; GHD 18% tested, of which 90% GHD	344 (W 130, M 214) 52 yr	General population	11.9 yr	All-cause mortality SMR: 2.17 (95% CI 1.88–2.51) W: 2.93 (95% CI 2.28–3.75), M: 1.91 (95% CI 1.59–2.28); CV mortality SMR: 1.75 (95% CI 1.40–2.19); CVA mortality SMR: 3.39 (95% CI 2.27–4.99) W: 4.91 (95% CI 2.62–8.40), M: 2.64 (95% CI 1.44–4.42); Cardiac mortality SMR: 1.41 (1.04–1.88)
Tomlinson et al. (2001) [10] UK	Hypopituitarism including 12% craniopharyngioma; Acromegaly + CD excluded; GHD 11% tested, of which 100% GHD	1014 (W 500, M 514) 45.3/46.2 yr	General population	8 yr	All-cause mortality SMR: 1.87 (95% CI 1.62–2.16) W: 2.29 (95% CI 1.75–3.00), M: 1.57 (95% CI 1.19–2.06); CV mortality SMR: 1.82 (*P* < 0.001); CVA mortality SMR: 2.44 (*P* < 0.001); Malignancy mortality SMR: 0.97 (*P* = 0.6)
Svensson et al. (2004) [20] Sweden	Hypopituitarism (no further data); Acromegaly + CD excluded; No GHD data	1411 (W 664, M 747) 56.9 yr	General population	5 yr	All-cause mortality SMR: 3.80 (95% CI 3.43–4.19) W: 4.54 (95% CI 3.89–5.26), M: 3.36 (95% CI 2.93–3.83); Malignancy mortality SMR: 3.92 (95% CI 3.21–4.76) W: 4.49 (95% CI 3.26–6.03), M: 3.59 (95% CI 2.74–4.63)

*Mean age given for total population or by sex

†Mean or median

CD: Cushing’s disease; CI: confidence interval; CV: cardiovascular; CVA: cerebrovascular accident; GHD: growth hormone deficiency; M: men; NR: not reported; SMR: standardized mortality ratio; W: women; yr: year

Bates et al. [7] described a standardized mortality ratio (SMR) of 1.73 (95% CI 1.28–2.18) in a cohort of 172 adult patients with hypopituitarism caused by a variety of etiologies. Age and hypogonadism were important influencing factors, with older and hypogonadal patients having lower mortality. This study also described a sex difference for all-cause mortality where women appeared to have a higher mortality (SMR 2.29, 95% CI 1.37–3.58) compared to men (SMR 1.50, 95% CI 1.02–2.13). Another, comparable Swedish study [6] demonstrated similar results with an increased overall mortality (SMR 2.17, 95% CI 1.88–2.51) in 344 patients with hypopituitarism excluding patients with prior Cushing’s disease or acromegaly. This study confirmed the higher mortality risk in women (SMR 2.93, 95% CI 2.28–3.75 in women vs. 1.91, 95% CI 1.59–2.28 in men). In particular, cerebrovascular mortality was increased in this cohort compared to the general population. For cerebrovascular mortality, women also had a greater risk of death (SMR 4.91, 95% CI 2.62–8.40) than men (SMR 2.64, 95% CI 1.44–4.42). Notably, almost all patients (86%) were treated with radiotherapy in addition to pituitary surgery.

In 2001, a large cohort study in 1014 patients with hypopituitarism by Tomlinson and colleagues [10] once again demonstrated an excess mortality, which also was significantly higher in women than in men. Due to the large cohort, they could perform sub-analyses, which showed an increased mortality risk for younger patients, patients with craniopharyngioma, patients treated with surgery or radiotherapy, and patients with hypogonadism. The authors argued against an important role for GHD in the increased mortality in hypopituitarism since the degree of hypopituitarism was not related to the risk of death in their study. However, GHD was not functionally tested in 89% of the patients and is known to be one of the first deficiencies to occur in pituitary insufficiency [21]. One of the last studies to describe mortality in hypopituitarism without the influence of GHRT is the study by Svensson and colleagues [20] in 2004. This study underlined the increased mortality risk as previously described. Of interest in their study was the relative high mortality risk due to malignancies (SMR 3.92, 95% CI 3.21–4.76). The authors hypothesized that the increased risk of death due to malignancies was caused by radiotherapy, the presence of multiple endocrine neoplasia syndrome, or GHRT use in childhood-onset hypopituitarism. Since no data was available on these characteristics, we cannot compare it to earlier studies.

When interpreting the above mentioned studies, one should realize that hypopituitarism and GHD are not synonymous. Severe GHD was not properly diagnosed in most patients and may not have been present in a small proportion of the cohorts. More recently, two studies investigating the long-term effect of GHRT have also presented mortality rates in accurately diagnosed, but untreated, GHD patients (control groups, not randomized) [18, 22]. The first to publish long-term data from a national registry on GHRT and mortality was van Bunderen and colleagues [18] in 2011. Their study contained a small (n = 109), non-randomized, control group of adult patients with severe GHD. The reason for not starting GHRT was related to a serious comorbidity (including malignancies and pituitary tumor recurrence) in only 12% of the patients. Mortality, in this small group of patients, was not significantly increased (SMR 1.42, 95% CI 0.79–2.56). In 2013, the US prospective, observational Hypopituitary Control and Complication Study (HypoCCS) [22] sponsored by Eli Lily and Company published data from their database, which included 442 untreated GHD patients with a mean follow-up of only 2.3 years. The frequency of mortality did not differ significantly (*P* = 0.73) between the treated and untreated group (1.66% vs. 2.49%) after adjusting for baseline differences. The SMR was not increased for the untreated GHD group (SMR 0.58, 95% CI 0.29–1.04).

**Fig. 1:**
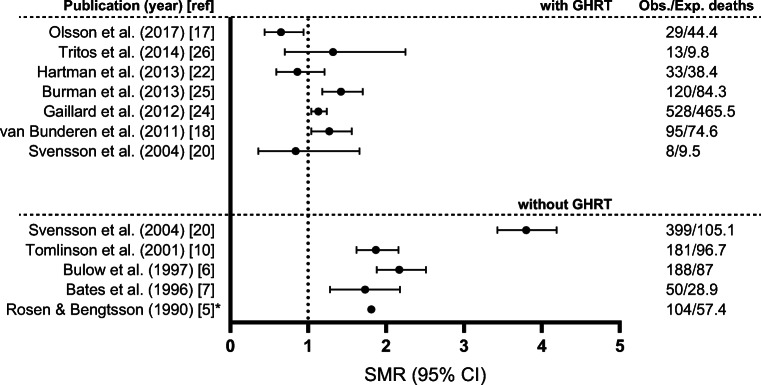
All-cause mortality in studies on hypopituitarism with or without growth hormone replacement therapy (GHRT). Data are given as standardized mortality ratios (SMRs) with 95% confidence intervals (CIs) and numbers of observed (Obs.) and expected (Exp.) deaths for studies with GHRT (upper) and without (lower) GHRT. *For Rosen & Bengtsson only the observed and expected number of deaths are given together with the ratio since no confidence interval was presented in this study

Interpreting the convincing excess mortality in the early cohort studies on hypopituitarism and the mortality rates in the later studies in patients diagnosed with GHD, one might notice a decreasing excess mortality over time (Fig. 1). This could be due to changes in other factors than GHD which can influence mortality such as better monitoring and treatment of the pituitary disorders and its co-morbidities as well as improved replacement therapy of the other anterior pituitary axes, since the latter studies were published more than two decades after the first study by Rosen and Bengtsson. However, the improvement in survival over recent decades seems to be small when looking at time trends of mortality in all patients with non-functioning pituitary adenoma regardless of hypopituitarism, in Sweden [23]. Of course, selection bias cannot be ruled out as it can influence the outcome in both directions. Therefore, the above mentioned studies, although to be interpreted with caution, do demonstrate a high likelihood for a role of GHD in the excess mortality demonstrated in adult patients with hypopituitarism.

## GHRT and mortality

In 2004, the previous mentioned study by Svensson and colleagues [20] was the first to describe mortality risk in a small cohort (n = 289) of properly diagnosed patients with severe GHD from a single hospital with a mean treatment duration of 5 years for GHRT. Mortality was not different from the general population (SMR 0.84, 95% CI 0.36–1.66). The authors suggested that GHRT reduced mortality in hypopituitary patients, since their hypopituitary cohort without GHRT had a highly increased risk of overall mortality (SMR 3.80, 95% CI 3.43–4.19). However, these two different cohorts were not fully comparable, e.g. patients in the hypopituitary cohort without GHRT was older at inclusion in the study compared to patients in the GHRT cohort.

Later, in 2011, van Bunderen and colleagues [18] published data on 2229 adult patients receiving GHRT from a national registry in the Netherlands. They demonstrated a slightly increased mortality compared to the background population (SMR 1.27, 95% CI 1.04–1.56), which was only increased in women (SMR 1.66, 95% CI 1.23–1.78) and not in men (SMR 1.06, 95% CI 0.81–1.40). The mortality, both in the overall cohort and in both sexes, was mostly due to cardiovascular diseases. Excluding patients with an inherent excess mortality risk (e.g. craniopharyngioma or possible malignant cause of hypopituitarism) normalized the mortality, which suggests that factors other than GHD/GHRT alone can influence mortality risk. After the exclusion of high-risk patients, the mortality risk due to cardiovascular disease remained increased for women (SMR 2.36, 95% CI 1.43–3.92). Gaillard and colleagues [24] reported the long-term effect of GHRT from the KIMS (Pfizer International Metabolic Database) database in 2012. They investigated all-cause and cause-specific mortality in 13,983 patients and also studied factors associated with mortality. In accordance with previous studies in hypopituitary patients, female sex, younger age at diagnosis, underlying diagnosis of craniopharyngioma or aggressive tumor, presence of diabetes insipidus, and pre-treatment with radiotherapy were all independently associated with increased mortality. All-cause mortality was elevated in women (SMR 1.56, 95% CI 1.36–1.78), but not in men (SMR 0.94, 95% CI 0.84–1.06). Subsequently, Burman et al. [25] investigated the causes of death in more detail among the 1286 Swedish patients registered in the KIMS database. They found an increased mortality, which was not only accounted for by an excess in cardiovascular mortality but also due to infections and suspected inadequately treated hypocortisolism during stress (based on a small number of patients, n = 8). In 2014, Tritos et al. [26] presented mortality data for a subgroup of patients from the KIMS database. They compared 164 patients with a diagnosis of GHD and acromegaly and 2467 patients with a diagnosis of GHD and non-functioning pituitary adenoma. The SMR in the GHD patients with acromegaly was 1.32 (95% CI 0.70–2.25). Surprisingly, the SMR was lower in GHD patients with a non-functioning pituitary adenoma than for the general population (SMR 0.58, 95% CI 0.48–0.94). The decreased mortality compared to the general population could be the effect of the close surveillance of these patients.

The sponsored prospective safety surveillance database, the HypoCCS, published their mortality data in 2013 [22]. They found a lower mortality in patients with GHRT compared to an untreated control group (not statistically significant after adjusting for baseline differences) and an SMR of 0.86 (95% CI 0.59–1.21) compared to the US general population, although the mean follow-up period was limited to 2.3 years. In 2014, Stochholm and colleagues (19) retrospectively studied the impact of socioeconomic factors on mortality in patients with adult GHD. Of the 276 patients, only 133 received GHRT and they had a decreased mortality risk compared to untreated patients (hazard ratio 0.34, 95% CI 0.15–0.77) even after adjustment for all measured confounders. Olsson and colleagues [17] studied a homogenous group of patients with only one underlying cause of GHD, a non-functioning pituitary adenoma. They found a significantly lower mortality (SMR 0.65, 95% CI 0.44–0.94) in the 207 patients treated with GHRT. This was also significantly lower compared to the control group of 219 patients with non-functioning pituitary adenoma who did not receive GHRT (not randomized). This was the first non-registry, single-center study to investigate GHRT in a single underlying etiology of GHD without an inherent excess mortality risk. Also, the follow-up duration was extensive, with a median GHRT duration of 12 years. Nevertheless, selection bias for those who received GHRT cannot be excluded. Table 2 shows an overview of the cited studies on GHRT and mortality.

**Table 2:** Overview of the articles published on mortality and growth hormone replacement therapy

Reference and country	Etiology	Study population (no.) and mean age*	Controls	Follow-up duration†	Conclusions
Svensson et al. (2004) [20] Sweden	Hypopituitarism including 4% craniopharyngioma and 5% extrapituitary tumor; Acromegaly + CD excluded	289 (F 103, M 186) 47.6 yr	General population	5 yr	All-cause mortality SMR: 0.84 (95% CI 0.36–1.66) W: 0 (95% CI 0.36–1.62), M: 1.11 (95% CI 0.48–2.20); CV mortality SMR: 0.27 (95% CI 0.03–0.99) W: 0 (95% 0–3.15), M: 0.33 (95% CI 0.04–1.18); CVA mortality SMR: 1.95 (95% CI 0.78–4.02) W: 1.22 (95% CI 0.02–6.80), M: 2.17 (95% CI 0.79–4.71); Malignancy mortality SMR: 0.88 (95% 0.35–1.80) W: 0.34 (95% CI 0.01–1.92), M: 1.17 (95% CI 0.43–2.56)
van Bunderen et al. (2011) [18] The Netherlands	Hypopituitarism including 11% craniopharyngioma, 9% extrapituitary tumor, and 10% acromegaly + CD	2229 (F 1069, M 1160) 42.6 yr	Untreated GHD (n = 109); General population	5.7 yr	All-cause mortality HR, untreated vs. treated: 1.89 (95% CI 0.86–4.13); All-cause mortality SMR: 1.27 (95% CI 1.04–1.56) W: 1.66 (95% CI 1.23–2.23), M: 1.06 (95% CI 0.81–1.40); CV mortality SMR: 1.35 (95% CI 0.95–1.94) W: 2.52 (95% CI 1.57–4.06), M: 0.84 (95% CI 0.49–1.45); Malignancy mortality SMR: 0.86 (95% CI 0.60–1.25) W: 1.12 (95% CI 0.66–1.88), M: 0.70 (95% CI 0.42–1.19)
Gaillard et al. (2012) [24] International (KIMS)	Hypopituitarism including 11% craniopharyngioma, 8% extrapituitary tumor, and 8% acromegaly + CD	13,983 (F 7174, M 6809) 43.8 yr	General population	4.9 yr	All-cause mortality SMR: 1.13 (95% CI 1.04–1.24) W: 1.56 (95% CI 1.36–1.78), M: 0.94 (95% CI 0.84–1.06); CV mortality SMR: 0.83 (95% CI 0.63–1.08); CVA mortality SM: 1.88 (95% CI 1.44–2.41); Malignancy mortality SMR: 0.88 (95% CI 0.74–1.03)
Burman et al. (2013) [25] Sweden (from KIMS)	Hypopituitarism including 11% craniopharyngioma, 10% extrapituitary tumor, and 7% acromegaly + CD; 99.7% GHRT use	1286 (F 612, M 674) 44.8 yr	General population	9.6 yr	All-cause mortality SMR: 1.42 (95% CI 1.18–1.70) W: 1.63 (95% CI 1.18–2.18), M: 1.33 (95% CI 1.05–1.66); CV mortality SMR: 1.21 (95% CI 0.81–1.74); CVA mortality SMR: 1.82 (95% CI 0.91–3.26); Malignancy mortality SMR: 0.92 (95% CI 0.61–1.34)
Hartman et al. (2013) [22] USA (HypoCCS)	Hypopituitarism including 11% craniopharyngioma	1988 (F 44%, M 56%) 46 yr	Untreated GHD (n = 442); General population	2.3 yr	All-cause mortality: 1.66% vs. GHD 2.49%; Malignancy mortality: 1.61% vs. GHD 2.71%; All-cause mortality SMR: 0.86 (95% CI 0.59–1.21)
Stochholm et al. (2014) [19] Denmark	Hypopituitarism including unknown number of craniopharyngioma, extrapituitary tumor, and acromegaly + CD	133 (F 72, M 61) 32 yr	Untreated GHD (n = 143); General population	15 yr	All-cause mortality HR: 1.78 (95% CI 0.84–3.77) vs. general population; All-cause mortality HR: 0.34 (95% CI 0.15–0.77) vs. untreated; Malignancy mortality HR: 3.45 (95% CI 1.09–10.93) vs. general population
Tritos et al. (2014) [26] International (KIMS)	GHD after acromegaly	164 (F 101, M 63 52.9 yr	NFPA (n = 2467); General population	5 yr	All-cause mortality SMR: 1.32 (95% CI 0.70–2.25) for acromegaly GHD vs. 0.58 (95% CI 0.48–0.70) for NFPA (*P* = 0.04); CV mortality SMR: 2.89 (95% CI 1.16–5.92) for acromegaly GHD vs. 0.68 (95% CI 0.48–0.94) for NFPA (*P* = 0.0004); CVA mortality SMR: 1.60 (95% CI 0.05–7.95) for acromegaly GHD 0.96 (95% CI 0.53–1.60) for NFPA (NS)
Olsson et al. (2017) [17] Sweden	NFPA	207 (F 62, M 145) 56.3 year	Untreated (n = 219) 11% (n = 25) tested for GHD, of which 100% GHD; General population	12 yr	A cox-model revealed a decreasing mortality with the years of GHRT (HR 0.94 [95% CI 0.91–0.98, *P* = 0.0063]) and an increasing mortality with age at start in the study (HR 1.10 [95% CI 1.08-1,12, *P* < 0.0001]). Other factors, such as treatment with radiotherapy, BMI, gender and different replacement therapies, did not have a significant effect. All-cause mortality SMR: 0.65 (95% CI 0.44–0.94) (*P* = 0.009 vs. untreated); W 0.71 (95% CI 0.31–1.41), M 0.63 (95% CI 0.39–0.97); Malignancy mortality SMR: 0.29 (95% CI 0.08–0.73)

*Mean age given for total population or by sex

†Mean or median

CD: Cushing’s disease; CI: confidence interval; CV: cardiovascular; CVA; cerebrovascular accident, GHD; growth hormone deficiency; GHRT; growth hormone replacement therapy; HR; hazard ratio; HypoCCS: Hypopituitary Control and Complication Study; KIMS: Pfizer International Metabolic Database; M: men; NFPA, non-functioning pituitary adenoma; NS: not statistically significant, ND: no data; SMR: standardized mortality ratio; W: women; yr, year

When interpreting the effect of GHRT on mortality (Fig. 1), one must remain critical. A number of prospective, randomized, placebo-controlled trials have suggested that, compared to placebo, GHRT has improved the detrimental effects of hypopituitarism attributed to GHD, but not in all [27]. This is in line with the results from many non-controlled retrospective and open-label observational studies [14], including most of the above-mentioned studies on survival. While placebo-controlled trials are challenging to complete in small patient populations, these trials nevertheless provide data with fewer confounders compared to open-label studies. Although comparisons with the background populations were corrected for age, sex, and calendar year in the mentioned retrospective cohort studies, an effect of factors other than GHRT cannot be ruled out. The association of mortality in hypopituitarism with confounding variables, such as younger age, sex, surgery or radiotherapy, or the underlying condition such as craniopharyngioma is also demonstrated in adults with GHD receiving GHRT. We earlier concluded that the increased mortality in hypopituitarism may be multifactorial and, therefore, not attributable to GHD alone. Thus, GHRT does not ameliorate all of the aspects of hypopituitarism/pituitary disease, but it does explains a proportion of the improved, but not always normalized, mortality risk.

## Shortcomings of studies investigating mortality in GHD and GHRT

Several aspects need to be considered when evaluating mortality in patients with GHD that have or have not received GHRT (Table 3). There is a large variety of different *underlying diseases causing hypopituitarism*. Some of these underlying diseases (e.g. non-functioning pituitary adenoma) entail no excess mortality, whereas some (e.g. craniopharyngioma) result in a large excess mortality no matter whether they have GHD with or without GHRT [9, 24, 28]. The large majority of studies investigating mortality in GHD patients with and without GHRT have a combination of patients with both low and high *inherent risk of excess mortality* [10, 18, 19], which will affect the outcome.

**Table 3:** Aspect that needs to be considered when evaluating studies on mortality in patients with GH deficiency that have or have not received GHRT

Factor	Example reference	Description
Risk of inherent excess mortality (underlying disease causing hypopituitarism)	Gaillard et al. [24]	Some of these underlying diseases (e.g. non-functioning pituitary adenoma) have no excess mortality, whereas some (e.g. craniopharyngioma) have a large excess mortality. In studies with a combination of patients with both low- and high-inherent risk of excess mortality, this factor will have an effect on the outcome.
Risk of inherent excess mortality (pituitary tumor treatments)	Stochholm et al. [19]	For example, treatment with radiotherapy can be related to excess mortality either because of a more severe underlying pituitary disease, needing a more aggressive tumor treatment, or due to side effects of the treatment itself.
Other aspects of hypopituitarism	Burman et al. [25]	Hypopituitarism often includes multiple deficiencies. Many of them, e.g. glucocorticoid replacement, can also affect the mortality in patients with hypopituitarism.
Potential surveillance bias	Olsson et al. [17]	The majority of all patients treated with GH are followed closely at specialized units. The close follow-up of these patients compared to the general population might be an advantage with respect to the development of (e.g. hypertension) or decreasing the frequency of negative behaviors (e.g. frequent alcohol intake).
Potential selection bias	Hartman et al. [22]	In observational cohort studies without randomization, there is always a potential selection bias.
Short follow-up/low number of events	Tritos et al. [26]	The follow-up needs to be sufficient to be able to predict mortality in a chronic treatment modality, like GHRT. In addition, few events in a study bring uncertainty to the results and makes it hard to properly control for multiple confounders.

GH: growth hormone; GHRT: growth hormone replacement therapy

A similar issue needs to be considered when investigating patients with different types of pituitary tumor treatments. For instance, treatment with radiotherapy can be related to an increased risk of death either because of a more severe underlying pituitary disease, needing a more aggressive treatment regime, or due to side effects of the treatment itself (for example, radiotherapy is associated with a risk of secondary brain tumors) [24, 29].

Pituitary diseases and its consequences, including hypopituitarism, lead to multiple treatment challenges. Many of them, such as glucocorticoid replacement, can also affect the mortality in patients with hypopituitarism [8]. In addition, almost all patients receiving GHRT are followed closely at specialized units that not only manage their GH substitution but also perform frequent overall health examinations. The close follow-up of these patients compared to the general population might be an advantage with respect to the development of, for example, type 2 diabetes mellitus or decreasing the frequency of negative behaviors, such as smoking. This could result in a *surveillance bias* for the group of patients with GHRT.

Furthermore, in some of the initial studies on mortality in patients with hypopituitarism and GHD, no formal *testing for severe GHD diagnosis* was performed in the majority of patients (5, 7, 10). Even though deficiency in the somatotropic axis is, in many cases, one of the first deficiencies in hypopituitarism, hypopituitarism and GHD are not synonymous [21]. This means that severe GHD might not have been present in all patients with hypopituitarism.

A more general issue that needs to be taken into account is the problem with *short follow-up, low number of events, and/or potential selection bias* in (retrospective) observational cohort studies. The follow-up needs to be sufficient to be able to predict the mortality in a chronic treatment modality, like GHRT [22]. Also, few events in a study brings uncertainty to the results and makes it impossible to properly control for multiple confounders [20]. Finally, there is always a potential selection bias in studies without randomization.

Considering all of the above-mentioned aspects (Table 3), the effect of each factor on outcome is hard to predict. Many of the studies on hypopituitarism and GHD or GHRT include patients with an inherent excess mortality risk which might overestimate mortality compared to a general population and underestimate the effect of GHRT. In studies on GHRT, potential selection bias, on the other hand, could underestimate the mortality risk.

## Future challenges and questions

### Future challenges

- No randomized trial has been performed to investigate either the effect of GHD or GHRT on mortality. Unfortunately, it would be an enormous task to perform such a study and it is questionable whether it would be ethical to withhold GHRT during long-term follow-up given the verified positive effects on aspects other than mortality.
- The beneficial effect of GHRT has been extensively demonstrated, but data in elderly patients with GHD is scarce [30]. It would be of interest to formally investigate the effects, including mortality, of long-term GHRT in older patients (over 60 years of age) with hypopituitarism and severe GHD.

### Questions

- The available evidence suggests that the mortality in adult patients with hypopituitarism including GHD is approaching normal in patients receiving modern replacement therapy including daily GHRT. Will this be similar for long-acting GHRT?
- Many of the studies investigating hypopituitarism including GHD as well as replacement with GH demonstrate a remarkable sex difference in (cardiovascular) mortality. What is the explanation for the higher (cardiovascular) mortality risk encountered in women compared to men?

## Conclusion

Several studies from the 1990s demonstrated an excess mortality in patients with hypopituitarism including a presumed or verified GHD. It was then suggested that the increased risk of death originated from the unreplaced GHD. Today, evidence suggests that the excess mortality in these patients has multiple causes, including not only the unreplaced GHD but also, for example, the etiology of hypopituitarism, non-physiological glucocorticoid replacement therapy as well as tumor treatment and its side effects.

The majority of studies on mortality in hypopituitary patients receiving GHRT show a normal risk of mortality compared to the background population. However, since the cause of the excess mortality in hypopituitary patients is multifactorial, the improvement also emanates from factors other than GHRT, such as advances in the treatment of other deficiencies and of the pituitary tumor.

The most important limitation of the literature in the field of survival in GHD patients with or without GHRT is the lack of prospective, randomized trials. Since a prospective, randomized trial would be a tremendous undertaking, and it could also be ethically questionable to let GHD patients go untreated for a long period of time, it is unlikely that we will ever see such a trial.

In conclusion, the evidence available to date suggests that GHD in patients with hypopituitarism contributes to an excess mortality and that modern replacement therapy including GHRT in these patients has resulted in a mortality that is approaching normal.
